# Reentrant phase behavior in systems with density-induced tunneling

**DOI:** 10.1038/s41598-024-60955-1

**Published:** 2024-05-06

**Authors:** A. Krzywicka, T. P. Polak

**Affiliations:** Institute of Spintronics and Quantum Information, Faculty of Physics, Adam Mickiewicz Univeristy in Poznań, Poznań, Poland

**Keywords:** Condensed-matter physics, Statistical physics, thermodynamics and nonlinear dynamics

## Abstract

We show that correlations in strongly interacting many-particle systems can create quantum decoherence, leading to a mechanism of dissipation that does not rely on an external source. Using analytical methods, we study a bosonic many body system in two dimensions, with extended interactions between particles. We show that, as expected, the system can be driven out of a coherent state. Surprisingly, when the interaction strength is sufficiently large, the system reenters the superfluid phase even after coherence is lost. The breakdown of quantum coherence is a certainty, but interpreting the process correctly relies on understanding and preserving the nature of the coupling between the constituents of the many particle system. The methods used provide a natural cutoff point at the critical temperature, where superfluidity breaks down.

## Introduction

The decoherence of quantum states due to coupling with external degrees of freedom has gained a lot of interest^1–8^. Especially enticing is the possibility of controlling how such systems dissipate energy. For instance, quantum information processing relies on precise control of non-classical states in the presence of many uncontrollable environmental degrees of freedom. Advancements in controlling quantum devices highlight the role of dissipation engineering in quantum error correction^9,10^. The mechanism of dissipation is embedded into some systems and thus impossible to avoid entirely, even when the external environment does not exist or can be sufficiently suppressed.

Dissipative behavior is usually generated by coupling the original system with external degrees of freedom. Driven-dissipative many body systems have been realized experimentally by coupling trapped ultra-cold atoms to the optical modes of a laser-driven atoms^1–6^. Dissipation can also be an effect of competition between different unitary Hamiltonian contributions of the system^3,5,8^. An increase of interest in theoretical descriptions of such systems has followed. Counterintuitively, within the right parameter range, dissipation can enhance coherence and entanglement^7,8,11–14^. This stabilization leads to a wealth of interesting phenomena, including emergent phase transitions, many body pair coherent states, and novel mode competition and symmetry breaking. In two-photon driven bosonic lattice models, the dissipative steady states can be found exactly^15^. A two-particle loss term can increase correlations to the point of effectively inhibiting dissipation altogether^16^. In high Tc superconductors, non-local dissipative bosonic mediators can act coherently and increase the superconducting critical temperature $T_{c}$^17^. The stabilizing effect of dissipation can also facilitate experimental observation of non-equilibrium and exotic states, such as superfluid time crystals^18–21^. Bosonic pairs, or doublons, have been studied in systems with loss, including three-body losses, which can be used to realize effective three-body interactions^22^. The complex nature of driven-dissipative many body models means that it is not possible to fully describe them using methods that do not account for quantum fluctuations and information on the spatial distributions of individuals^15,23^. Therefore, up to now, the body of work has consisted mainly of relatively limited approaches, such as few-body systems and one-dimensional studies^24^. Furthermore, for all the new and interesting phenomena that have already been observed, dissipation has consistently been treated as an external factor. In this work, we focus on a different facet of dissipative behavior: one that is an implicit property of a strongly correlated model with extended interactions. It is known that dissipation can generate effective many body interactions; we show that the opposite is also possible: many body interactions can themselves be a source of dissipative behavior.

The generic Bose-Hubbard model (BHM) for strongly interacting bosons has been studied using a plethora of methods and approaches^25–32^. Its extended versions are much more laborious to analyze and therefore less abundant. It is not enough to study simplifications of extended BHMs, as those cannot describe quantum fluctuations, especially in lower dimensions^33–39^. Failure to capture the long length scale averages of order parameters near critical points leads to unphysical phase transitions for arbitrary chosen densities. The complications extend to experiments: pure density-induced tunneling is difficult to explicitly replicate in bulk materials, due to its complexity and lack of control over experimental parameters. Nevertheless, strong interactions always introduce multi-particle correlations that exist locally throughout the entire considered system. Therefore, many body correlations are always present in optical lattice systems, even if only the standard Hamiltonian is used to analyze experimental data^40,41^. Whatever methods are used should not exclude correlations from the start.

The main motivation of the presented work are both previous theoretical considerations and experimental data. The behavior of the condensate is complex at low temperatures, because of intricate interactions that come into play in such conditions. A depletion (the existence of a finite non-condensed fraction) arises from quantum fluctuations and affects the coherence. Quantum fluctuations generate interactions which are not present explicitly in the model, such as the density induced interaction. The latter can be derived separately, as an extended version of the generic Bose-Hubbard Hamiltonian. Such interactions are usually understood as factors which merely support the coherence of the condensed fraction of atoms^37^. However, that is only true when the approach excludes quantum fluctuations.

Path integrals used in this paper constitute a flexible framework beyond the limitations of those simpler approaches that fail to reproduce quantum fluctuations of collective motion. The quantum rotor method shifts the correlations between particles from bosonic fields *b* to phase fields $\phi$, facilitating analytical study of the critical behavior of strongly-correlated many body systems. These methods allow to observe that pair condensation occurs implicitly within the density-induced tunneling (DIT) BHM^42^. It is represented by a double cosine term in the effective phase action. Double cosine terms can be further linked to dissipative behavior^43^. The pairing term that emerges from density-induced tunneling can therefore be treated as dissipative. We study the effect such dissipation has on the single-particle superfluid.

We consider two versions of a pairing-based dissipative model derived from the DIT BHM. One is assumed as a coupling between the standard single condensate and the pair condensate, which is treated as an external source^44^. The other version is derived directly from the imaginary time–dependent effective phase model and contains an intrinsic dissipative term. We compare the critical lines of the two systems and study the effect of the pair condensate on the single condensate at different particle densities. We observe a revival of the single superfluid as the DIT coefficient, which generates the pairing mechanism and therefore the dissipative behavior, increases.

We would like to emphasize the difference between our approach and previous ones, including several types of dissipation–like processes that can be found in the literature. The dissipative, or open, quantum system consists of two elements: a main, closed system is coupled to an often (but not necessarily) larger classical or quantum system. The environment can be assumed to have Markovian-like properties; its dynamics can be described using the master equation in its Lindblad form^45–48^. The latter strongly depends on the nature of the coupling between systems. This approach is very prolific, since it can be applied to various experimentally relevant processes, depending on the form of the Lindblad operator. Importantly, although the Lindblad operator conserves the total particle number, it destroys coherence throughout the entire system. A quantitative determination of the effects of dissipation in many body systems is possible only in terms of a fully quantum mechanical description of the model^44,49^. All the environmental modes which give rise to relevant dissipation mechanisms have to be included in such considerations. A common practice is to assume the nature of the coupling; environmental modes are usually represented as harmonic oscillators with a continuously distributed resonant frequency. One version of our calculations makes use of this approach; we show its benefits and drawbacks. In the literature, the coupling which provides dissipative effects is always assumed in the microscopical model and any related macroscopic phenomena emerge from that assumption. We derive dissipative behavior from within the microscopic model itself. We explore how the condensate might be affected by an intrinsic type of dissipation, as opposed to one inserted into the Hamiltonian via a separate, external term.

The closest experimental setup to realize this model can be constructed within optical lattices and has in fact already been realized^50^, including liquid Helium experiments^51,52^. Considerable interest since the realization of atomic condensates, especially in the context of quantum depletion, has led to counterintuitive observations in systems of differing interaction strength and gas dilution^53,54^. In systems with strong interactions, the depletion is large in comparison with weakly-interacting diluted quantum gases. In experiments with liquid Helium, the fraction of correlated particle pairs coexists with the ground state of macroscopically occupied condensate. Furthermore, in the high density and strongly-interacting regime, pairs with anti-correlated momenta were detected^50^.

We derive a system of relatively low densities and strong interactions. Experimental realization would require control not only of the extended interaction, but also of the amount of energy required to add or subtract particles from the system: and therefore the density of the particles. We show that suppressing density fluctuations is the key starting point to implementing this extended Bose-Hubbard model in experiments. Our calculations can help understand how the condensate is affected by correlations that emerge from the system itself in experiments with strongly-interacting Bose atoms. We thus provide a natural explanation of coherence loss within quantum systems, which are not necessarily connected with external degrees of freedom, but themselves generate such dissipative environments.

## Model

The theoretical description of strongly interacting bosons placed in a two-dimensional square lattice starts with the Bose-Hubbard model (BHM) with density-induced tunneling (DIT):

1 $$\begin{aligned} \hat{H}&= \frac{U}{2}\sum _{i}\hat{n}_{i}\left( \hat{n}_{i}-1\right) -\frac{1}{2}t\sum _{\left\langle i,j\right\rangle }\hat{a}_{i}^{\dagger }\hat{a}_{j}-\mu \sum _{i}\hat{n}_{i}+\nonumber \\&\quad -T\sum _{\left\langle i,j\right\rangle }\hat{a}_{i}^{\dagger }\left( \hat{n}_{i}+\hat{n}_{j}\right) \hat{a}_{j}+c.c., \end{aligned}$$

with on-site interaction *U*, nearest-neighbor tunneling *t*, chemical potential $\mu$ and density-induced tunneling *T*. We concentrate on the low temperature limit, and low densities. These assumptions surface naturally during analysis and will be explained further as they become relevant. In low temperatures, three phases can be recognized in this system: the Mott insulator phase, in which particles occupy lattice sites evenly and coherence is lost, the single particle superfluid and the pair superfluid. We focus specifically on the impact of the pair condensed fraction on coherence in the single particle condensed phase.

## Method

The quantum rotor (QR) analysis, used to prevent the $\textrm{U}\left( 1\right)$ symmetry of the variables, is divided into two parts. First, two sets of coefficients are determined for the effective phase model. Further treatment is the same for both options: the obtained phase model is mapped onto the quantum rotor model. This method reduces the problem of calculating critical lines to finding the saddle point of the rotor constraint. To concentrate on the changes resulting from analyzing a new physical system, all unnecessary details of the calculations are omitted. It is worth emphasizing that although the approach is known, its application to a new Hamiltonian is rather challenging, since the model is complex and its critical properties are governed by the preserved phase correlations.

### $\textrm{U}\left( 1\right)$ description of the model

Using the QR method within the path integral framework, the DIT BHM can be rewritten as a phase model^42^. This requires gauge transformation, which introduces the phase field $\phi$ and changes the bosonic variables:

2 $$\begin{aligned} a_{i}\left( \tau \right)&=e^{i\phi _{i}\left( \tau \right) }b_{i}\left( \tau \right) ,\end{aligned}$$

3 $$\begin{aligned} \bar{a}_{i}\left( \tau \right)&=e^{-i\phi _{i}\left( \tau \right) }\bar{b}_{i}\left( \tau \right) . \end{aligned}$$

A $4\times 4$ Nambu-like space is also introduced, in order to express the amplitudes *b* in terms of a Gaussian integral. After integration, the partition function of the obtained phase-only model is:

4 $$\begin{aligned} \mathcal {Z}=&\int \mathcal {D}\phi \,e^{-\mathcal {S}\left[ \phi \right] }, \end{aligned}$$

with effective action

5 $$\begin{aligned} \mathcal {S}\left[ \phi \right]&=\int _{0}^{\beta }d\tau \,\left\{ \sum _{i}\frac{1}{2U}\left[ \dot{\phi }_{i}\left( \tau \right) \right] ^{2}+\sum _{i}\frac{\tilde{\mu }}{iU}\dot{\phi }_{i}\left( \tau \right) -\text {Tr}\ln \Gamma \right\} , \end{aligned}$$

where the $\Gamma$ matrix has the form:

6 $$\begin{aligned} \Gamma =\left( \begin{array}{cccc} 0 &{} \frac{1}{2}\delta _{ij}\Delta _{i} &{} \frac{1}{2}\left( G_{0}^{-1}+S_{ij}\right) &{} 0\\ \frac{1}{2}\delta _{ij}\bar{\Delta }_{i} &{} 0 &{} 0 &{} 0\\ 0 &{} 0 &{} 0 &{} \frac{1}{2}\delta _{ij}\Delta _{i}\\ 0 &{} \frac{1}{2}\left( G_{0}^{-1}+S_{ij}\right) &{} \frac{1}{2}\delta _{ij}\bar{\Delta }_{i} &{} 0 \end{array}\right) \end{aligned}$$

and parameters present within are:

7 $$\begin{aligned} G_{0}^{-1}&=\left( \frac{\partial }{\partial \tau }+\bar{\mu }\right) , \end{aligned}$$

8 $$\begin{aligned} S_{ij}=&-\left( t-2T\right) e^{-i\phi _{ij}\left( \tau \right) }-\frac{4\bar{\mu }}{U}Te^{-i\phi _{ij}\left( \tau \right) }+\nonumber \\&-\frac{8}{U}T^{2}e^{-i2\phi _{ij}\left( \tau \right) }\cdot \left( 4\left\langle \bar{b}_{i}b_{j}\right\rangle +\delta _{ij}\right) ,\end{aligned}$$

9 $$\begin{aligned} \Delta _{i}&=-\frac{8}{U}T^{2}e^{-i2\phi _{ij}\left( \tau \right) }\left\langle b_{i}b_{i}\right\rangle ,\end{aligned}$$

10 $$\begin{aligned} \bar{\Delta }_{i}&=-\frac{8}{U}T^{2}e^{-i2\phi _{ij}\left( \tau \right) }\left\langle \bar{b}_{i}\bar{b}_{i}\right\rangle , \end{aligned}$$

where the chemical potential has been shifted to

11 $$\begin{aligned} \bar{\mu }&=\mu +\frac{U}{2}. \end{aligned}$$

This phase model corresponds exactly to the DIT BHM, with no approximations required. Following standard procedure, the next step is simplifying the effective action into a manageable form. The trace of the Green’s function can be rewritten and approximated by $\ln \left( 1+x\right) \approx x$, resulting in

12 $$\begin{aligned} \textrm{Tr}\ln \Gamma ^{-1}&\approx G_{0}^{2}\left[ \bar{\Delta }_{i}\Delta _{i}-S_{ij}^{2}\right] +2S_{ij}G_{0}. \end{aligned}$$

Calculating the bosonic averages in Eqs. (8–10) and transforming $G_{0}$ into a more useful form completes the analytical transformation. The effective phase action in its final form,

13 $$\begin{aligned} \mathcal {S}\left[ \phi \right] =\mathcal {S}_{\textrm{U}}\left[ \phi \right] +\mathcal {S}_{1}\left[ \phi \right] +\mathcal {S}_{\textrm{2}}\left[ \phi \right] , \end{aligned}$$

is comprised of three parts: an interaction part,

14 $$\begin{aligned} \mathcal {S}_{\textrm{U}}\left[ \phi \right] =\frac{1}{2U}\sum _{\left\langle i,j\right\rangle }\int _{0}^{\beta }d\tau \left( \frac{\partial \phi _{i}}{\partial \tau }\right) ^{2}+\frac{\bar{\mu }}{iU}\dot{\phi }_{i}\left( \tau \right) , \end{aligned}$$

a single condensation part,

15 $$\begin{aligned} \mathcal {S}_{1}\left[ \phi \right] =g_{1}\sum _{\left\langle i,j\right\rangle }\int _{0}^{\beta }d\tau \cos \left[ \phi _{i}\left( \tau \right) -\phi _{j}\left( \tau \right) \right] , \end{aligned}$$

and a pair condensation part,

16 $$\begin{aligned} \mathcal {S}_{\textrm{2}}\left[ \phi \right] =g_{2}\sum _{\left\langle i,j\right\rangle }\int _{0}^{\beta }d\tau d\tau ^{'}\,\cos 2\left[ \phi _{i}\left( \tau \right) -\phi _{j}\left( \tau ^{'}\right) \right] . \end{aligned}$$

The condensate coefficients $g_{1}$ and $g_{2}$ depend on the treatment of $G_{0}$. We consider two possible approaches. The simpler option is to approximate $G_{0}$. Deriving the coefficients explicitly using Eq. (7) is more complicated, but we find that doing so reveals implicit dissipative behavior contained within the model.

#### Effective amplitudes

The traditional approach is to approximate $G_{0}$ by $b_{0}^{2}$, which is obtained by minimizing the Hamiltonian^55,56^:

17 $$\begin{aligned} \frac{\partial }{\partial b_{0}}\left. \mathcal {H}\right| _{b=b_{0}}=0. \end{aligned}$$

In the case of the DIT BHM Hamiltonian, Eq. (1),

18 $$\begin{aligned} b_{0}^{2}=\frac{z\left( t-4T\right) +\left( \frac{U}{2}+\mu \right) }{U-8zT}. \end{aligned}$$

This approximation has been deemed sufficient to study low-temperature effects. For correlations in the $\varvec{k}$ space, it can be extended using e.g. the Bogoliubov approach^57^. Atom-atom correlations and time of flight images can thus be obtained in optical lattice systems within one consistent theory.

The single and pair condensation coefficients, respectively, are as follows:

19 $$\begin{aligned} g_{1}&= -\frac{z\left( t-4T\right) +\left( \frac{U}{2}+\mu \right) }{U-8zT}\left( 2\left( t-2T\right) +\frac{8\bar{\mu }}{U}T\right) \nonumber \\&\quad +\left[ \frac{z\left( t-4T\right) +\left( \frac{U}{2}+\mu \right) }{U-8zT}\right] ^{2}\left( \frac{64\bar{\mu }}{U^{2}}T^{3}+\frac{16}{U}JT^{2}\right) \nonumber \\&\quad \times \left\{ 2\left[ \coth \left( -\frac{\beta \mu }{2}\right) +\coth \left( \frac{\beta \left( \mu +U\right) }{2}\right) \right] +1\right\} ,\end{aligned}$$

20 $$\begin{aligned} g_{2}&= \left( \frac{z\left( t-4T\right) +\left( \frac{U}{2}+\mu \right) }{U-8zT}\right) ^{2}\nonumber \\&\quad \times \left[ \left( t-2T\right) ^{2}+\left( \frac{4\bar{\mu }}{U}T\right) ^{2}+2\left( t-2T\right) \frac{8\bar{\mu }}{U}T\right] . \end{aligned}$$

These amplitudes are known to provide adequate results in the study of low temperature properties, e.g., to analyze the thermodynamical functions and recover the well known $\lambda$ peaks in the specific heat, which signal single and pair condensation phases of matter^42^.

#### Derived model

We introduce an alternative, more robust approach: keeping the original, imaginary time–dependent form of $G_{0}$, Eq. (7) which after Fourier transform takes the form of

21 $$\begin{aligned} G_{0}=\frac{-i\omega _{m}+\bar{\mu }}{\omega _{m}^{2}+\bar{\mu }^{2}}. \end{aligned}$$

The condensate coefficients depend on imaginary time, providing new physical effects:

22 $$\begin{aligned} g'_{1}\left( \omega _{m}\right)&= -\frac{-i\omega _{m}+\bar{\mu }}{\omega _{m}^{2}+\bar{\mu }^{2}}\left( 2\left( t-2T\right) +\frac{8\bar{\mu }}{U}T\right) \end{aligned}$$

23 $$\begin{aligned}&\quad +\frac{1}{\left( -i\omega _{m}+\bar{\mu }\right) ^{2}}\left( \frac{64\bar{\mu }}{U^{2}}T^{3}+\frac{16}{U}JT^{2}\right) \end{aligned}$$

24 $$\begin{aligned}&\quad \times \left\{ 2\left[ \coth \left( -\frac{\beta \mu }{2}\right) +\coth \left( \frac{\beta \left( \mu +U\right) }{2}\right) \right] +1\right\} ,\end{aligned}$$

25 $$\begin{aligned} g'_{2}\left( \omega _{m}\right)&= \frac{1}{\left( -i\omega _{m}+\bar{\mu }\right) ^{2}}\nonumber \\&\quad \times \left[ \left( t-2T\right) ^{2}+\left( \frac{4\bar{\mu }}{U}T\right) ^{2}+2\left( t-2T\right) \frac{8\bar{\mu }}{U}T\right] . \end{aligned}$$

In this version, imaginary time–dependent terms are present in both condensation parts of the effective phase model, $\mathcal {S}_{1}$ and $\mathcal {S}_{\textrm{2}}$. The single coefficient $g'_{1}$ generates two contributions, one of which has an additional dissipation-like impact, Eq. (22). However, this term depends on higher orders of *T*/*U* than $g'_{2}$, so at $T/U\ll 1$ the pair dissipation is much stronger. The second Eq. (23), is negligible in low temperatures after Matsubara summation. Therefore, in this work, we forgo the marginally relevant contributions introduced by the single condensation coefficient $g'_{1}$ and replace it with the approximated $g_{1}$ of Eq. (19), focusing on the properties of the pair term, Eq. (16), in low temperatures.

The effective action is much the same as in the simpler model, Eq. (13), the only difference being that $\mathcal {S}_{\textrm{2}}$ is now explicitly dissipative:

26 $$\begin{aligned} \mathcal {S}'_{\textrm{2}}\left[ \phi \right] =g'_{2}\sum _{\left\langle i,j\right\rangle }\int _{0}^{\beta }d\tau d\tau ^{'}\,\frac{1}{\left( \tau -\tau ^{'}\right) ^{2}}\cos 2\left[ \phi _{i}\left( \tau \right) -\phi _{j}\left( \tau ^{'}\right) \right] , \end{aligned}$$

where

27 $$\begin{aligned} g'_{2}=\left( t-2T\right) ^{2}+\left( \frac{4\bar{\mu }}{U}T\right) ^{2}+2\left( t-2T\right) \frac{8\bar{\mu }}{U}T \end{aligned}$$

is the derived pair condensate coefficient.

### Dissipative phase models

In many body effective phase models, dissipative terms are proportional to $\left( \tau -\tau ^{'}\right) ^{-2}$. Traditionally, those terms are added to the Hamiltonian as arbitrary external factors. In this model, however, the microscopic Hamiltonian already contains the relevant term. Both versions of the pair condensation part of the effective action can be rewritten as dissipative. Pair condensates have been shown to exhibit dissipative behavior in experiments^50,51,54^, causing single condensate depletion.

Since the action derived from Matsubara time contains full information about quantum fluctuations, the dissipative nature of the pair condensate emerges naturally in Eq. (26). After series expanding the double cosine, we rewrite the derived pair effective action term $\mathcal {S}'_{\textrm{2}}$ as an explicitly dissipative term:

28 $$\begin{aligned} \mathcal {S}'_{\textrm{2}}\left[ \phi \right] =2g'_{2}\sum _{\left\langle i,j\right\rangle }\int _{0}^{\beta }d\tau d\tau ^{'}\,\frac{1}{\left( \tau -\tau ^{'}\right) ^{2}}\left[ \phi _{i}\left( \tau \right) -\phi _{j}\left( \tau ^{'}\right) \right] ^{2}. \end{aligned}$$

In the simpler version of the model, based on Eq. (18), the imaginary time factor does not emerge naturally. To study the dissipative effect of the pair term in Eq. (16), we treat the two condensates as separate, harmonically coupled systems: condensed bosons submerged in a bath of harmonic potential, created by the pair condensed system. The derivation of the effective action is typical for such many body systems and has been carried out under various circumstances^49^. The double cosine action term in Eq. (16) is then transformed into a dissipative term:

29 $$\begin{aligned} \mathcal {S}_{\textrm{2}}\left[ \phi \right] =2g_{2}\sum _{\left\langle i,j\right\rangle }\int _{0}^{\beta }d\tau d\tau ^{'}\,\left[ \frac{\phi _{i}\left( \tau \right) -\phi _{j}\left( \tau ^{'}\right) }{\tau -\tau ^{'}}\right] ^{2}. \end{aligned}$$

Ultimately, the two approaches differ only by their pair condensate coefficients:

30 $$\begin{aligned} G_{0}\begin{array}{c} \nearrow \\ \searrow \end{array}\begin{array}{c} b_{0}\begin{array}{cc} \text {coupled condensates} &{} \begin{array}{l} \rightarrow g_{1}\text { single particle}\\ \rightarrow g_{2}\text { pair } \end{array}\end{array}\\ \\ G_{0}\begin{array}{cc} \text {full treatment} &{} \begin{array}{cl} \rightarrow g'_{1} &{} \rightarrow g_{1}\text { single particle}\\ &{} \rightarrow g'_{2}\text { pair } \end{array}\end{array} \end{array} \end{aligned}$$

At a glance, the difference is trivial, but the two models exhibit substantially distinct behavior, as shown in the “Results” section. The proper treatment of quantum fluctuations requires an understanding of the properties of the derived actions, as well as the application of relevant approximations, which are different for the assumed and the derived model.

### Quantum rotor model mapping

The two models presented in the previous section describe the same phenomenon, but emerge from different interactions in two different systems, and as such are ruled by different pair coefficients $g_{2}$. However, the distinction only becomes relevant after the critical line equation has been derived. Thus, in the following section, both versions of the dissipative phase model can be treated identically, as one. The effective phase model is mapped onto the quantum rotor model. The free energy of the latter is then minimized with use of the saddle point method, in order to obtain the critical line equation. Only at this point do the two versions require separate treatment.

The Fourier-transformed quantum rotor partition function is

31 $$\begin{aligned} \mathcal {Z}=\int _{-i\infty }^{+i\infty }\left[ \prod _{i}\frac{\mathcal {D}\lambda \left( \tau \right) }{2\pi i}\right] e^{-N\phi \left[ \lambda \right] }, \end{aligned}$$

where

32 $$\begin{aligned} \phi \left[ \lambda \right] =-\beta \lambda -\frac{1}{2N}\sum _{k}\ln \left\{ \frac{1}{\beta \pi }\left[ \lambda -g_{1}\xi _{k}+\mathcal {G}^{-1}\left( \omega _{m}\right) \right] \right\} , \end{aligned}$$

with Lagrange multiplier $\lambda$ and lattice constant $\xi _{k}=2\sum _{d}\cos k_{d}$.

The critical line equation is derived by minimizing free energy with respect to the rotor constraint $\lambda$:

33 $$\begin{aligned} \frac{\partial \mathcal {F}}{\partial \lambda }=0. \end{aligned}$$

After rewriting lattice dependence in terms of the density of states function, defined as

34 $$\begin{aligned} \rho \left( E\right) =\frac{1}{N}\sum _{k}\delta \left( E-\xi _{k}\right) , \end{aligned}$$

the critical line equation is

35 $$\begin{aligned} 1=\frac{1}{2\beta }\int dE\,\sum _{m}\frac{\rho \left( E\right) }{\lambda -g_{1}E+\mathcal {G}^{-1}\left( \omega _{m}\right) }, \end{aligned}$$

where

36 $$\begin{aligned} \mathcal {G}\left( \tau ,\tau ^{\prime }\right) =\exp \left[ \frac{1}{\beta }\sum _{m}\frac{1-\cos \left[ \omega _{m}\left( \tau -\tau ^{\prime }\right) \right] }{\frac{1}{2U}\omega _{m}^{2}+4g_{2}\left| \omega _{m}\right| }\right] \end{aligned}$$

is the phase-phase correlator. In low temperatures and densities $\mu /U<\left( 1-\sqrt{3}\right) 2$, the inverse of $\mathcal {G}$ can be approximated by

37 $$\begin{aligned} \mathcal {G}^{-1}\left( \tau ,\tau ^{\prime }\right) \approx \frac{1}{2U}\omega _{m}^{2}+4g_{2}\left| \omega _{m}\right| . \end{aligned}$$

At the critical point, the Lagrange multiplier $\lambda$ can be substituted by its saddle-point value, $\lambda _{0}=g_{1}\xi _{max}$. The critical line equation, after performing Matsubara summation in low temperatures $\beta \rightarrow \infty$ limit, is then

38 $$\begin{aligned} 1&= \frac{1}{2\pi }\int d\xi \,\frac{\rho \left( \xi \right) }{g\left( \xi \right) }\left\{ \psi ^{(0)}\left[ \frac{\beta U}{\pi }\left( 4g_{2}+g\left( \xi \right) \right) \right] \right. \nonumber \\&\quad \left. -\psi ^{(0)}\left[ \frac{\beta U}{\pi }\left( 4g_{2}-g\left( \xi \right) \right) \right] \right\} , \end{aligned}$$

where

39 $$\begin{aligned} g\left( \xi \right) =\sqrt{\left( 4g_{2}\right) ^{2}-2\frac{g_{1}}{U}\left( \xi _{max}-\xi \right) } \end{aligned}$$

and $\psi ^{(0)}$ are digamma functions. The critical Eq. (38) is the second pivotal point in this analysis. It contains all information about the system, including explicitly the geometry of the bipartite lattices, here two-dimensional square. In the low temperature limit, digamma functions can be approximated as logarithms, leading to the final form of the critical line equation:

40 $$\begin{aligned} 1&=\frac{1}{2\pi }\int d\xi \,\frac{\rho \left( \xi \right) }{g\left( \xi \right) }\ln \left[ \frac{4g_{2}+g\left( \xi \right) }{4g_{2}-g\left( \xi \right) }\right] . \end{aligned}$$

Although this analysis is constrained to the low temperature limit, it can be expanded for finite temperatures, as well as different geometries.

## Results

**Figure 1:**
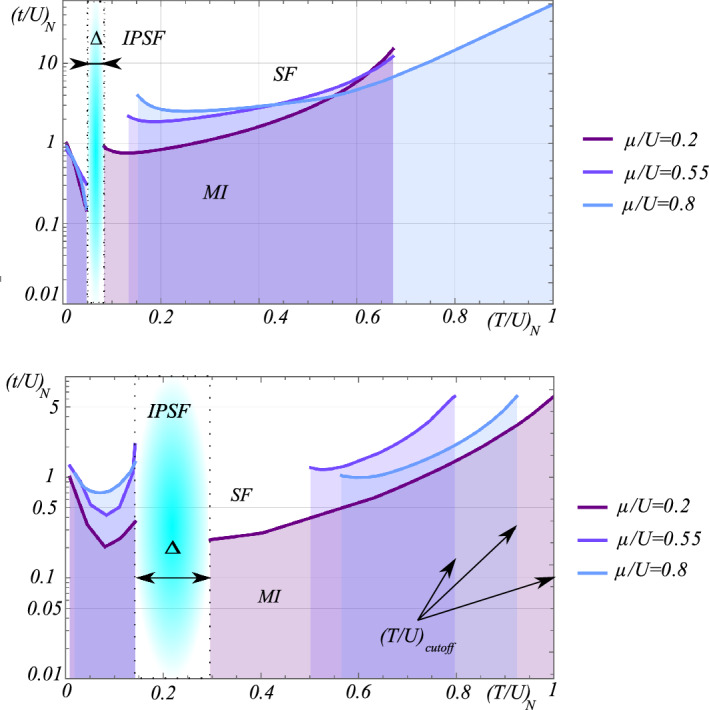
Comparison of the dependence of normalized single hopping $\left( t/U\right) _{N}$ on the normalized (see definition in text) DIT coefficient $\left( T/U\right) _{N}$ at different chemical potentials $\mu /U$. Top: approximated model, using $g_{2}$, Eq. (20). Bottom: derived model, using $g'_{2}$, Eq. (27). The critical lines separate the superfluid SF (above) and the Mott insulator (below) phases. The incoherent pair superfluid phase (IPSF) occurs in the gap region $\Delta$.

This work focuses on low temperatures, $\beta \rightarrow \infty$, and low density systems, $\mu /U<\left( 1+\sqrt{3}\right) /2$, as the essential phenomena take place within this parameter space. For more information about the range of chemical potential explored in this work, see Appendix 1. We focus on the low temperature region. As we see in Fig. 1, we are below the critical value of the nearest neighbor tunneling parameter in the density induced hopping region. The resulting critical lines do not extend far beyond $(t/U)_{\textrm{crit}}$, especially when the full treatment is applied. Therefore, suitable conditions for observing the emerging physical effects should be possible to attain within existing experimental setups, at temperatures below critical for the condensate. Exemplary critical lines determined by Eq. (40), which separate the Mott insulator (MI) and superfluid (SF) phases of the single particle condensate, are shown in Fig. 1. The proper energy scale of the system must be determined. Both nearest-neighbor tunneling *t*/*U* and density-induced tunneling *T*/*U* have been normalized by the critical value $(t/U)_{\textrm{crit}}$,

41 $$\begin{aligned} \frac{t/U}{(t/U)_{\textrm{crit}}}&\rightarrow \left( t/U\right) _{N}, \end{aligned}$$

42 $$\begin{aligned} \frac{T/U}{(t/U)_{\textrm{crit}}}&\rightarrow \left( T/U\right) _{N}, \end{aligned}$$

which separates the MI and SF phases in the absence of the extended interaction. The quantity *t*/*U* is calculated directly from Eq. (40) for given values of chemical potential and density induced tunneling. The presented theory contains no independent parameters. We also introduce the cutoff parameter, $\left( t/U\right) _{cutoff}$, which is connected with the critical properties of the correlation function. The physical origin of the cutoff is the suppression of both superfluids, single and pair, by quantum fluctuations. For ease of presentation, we also define a gap parameter, $\Delta$, which describes the region of incoherent pair superfluid. Within this region, the coherence of the single superfluid is lost, meaning there is no particle mobility: $\left( t/U\right) _{N}\rightarrow 0$. However, an incoherent fraction of pair superfluid remains. The rapid decrease of the normalized hopping $\left( t/U\right) _{N}$ is associated with two mechanisms. The first stems from the low temperature properties of the phase-phase correlation function (Appendix 1). A series expansion around the critical point shows that the density-induced interaction *T* both linearly suppresses the hopping amplitude and supports particle mobility for $\left( t/U\right) _{N}$ with larger powers of $\left( T/U\right) _{N}$:

43 $$\begin{aligned} \left( t/U\right) _{N}&\simeq 1-4\sqrt{2\pi }(4\mu /U+1)\left( T/U\right) _{N}\nonumber \\&\quad +48\pi \left( 2\mu /U+1\right) ^{2}\left( T/U\right) _{N}^{2}\nonumber \\&\quad -\frac{1}{2}\left( 48\pi \right) ^{2}\left( 2\mu /U+1\right) ^{4}\left( T/U\right) _{N}^{4}\nonumber \\&\quad +\frac{1}{2}\left( 48\pi \right) ^{3}\left( 2\mu /U+1\right) ^{6}\left( T/U\right) _{N}^{6}+\ldots \end{aligned}$$

These two effects interchange with increasing powers of the expansion which can be missed using premature cutoff. The second decreasing mechanism stems from the $\textrm{U}\left( 1\right)$ approach providing complete suppression of particle mobility; this effect cannot be analytically derived from the critical properties of the Eq. (40). A sudden revival of the coherent phase is also observed. As the density tunneling term increases, the quantum fluctuations reestablish long range order within the system, up until rapid cutoff at $\left( T/U\right) _{cutoff}$. Beyond this cutoff, the correlation function diverges. Therefore, this theory cannot account for larger values of density-induced tunneling *T*. At first glance, the results from both models in Fig. 1 seem almost identical; the differences are clarified further on.

**Figure 2:**
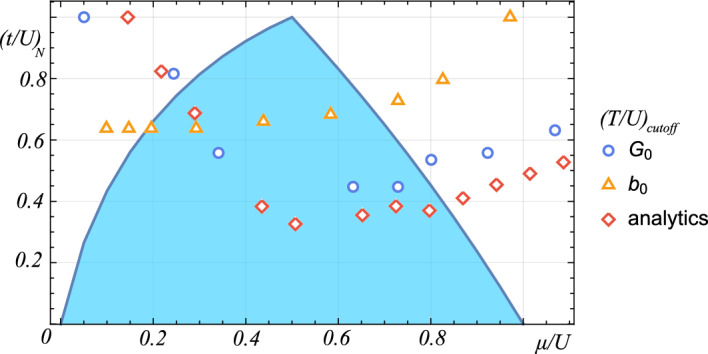
Top: cutoff values of $(T/U)_{N}$ calculated numerically using assumed model (orange triangles), derived model (blue circles) and analytics, compared to the first lobe of the zero-temperature square lattice superfluid (above)–Mott insulator (below) phase diagram (blue line).

The behavior of the revival showcases the most important difference between the assumed and derived models, presented in Fig. 2 in comparison to analytical results. Although the assumption made in the simplified model about the harmonic coupling between two condensates is reasonable and provides a qualitatively good description of the behavior of the system, it fails to reproduce the disappearance of coherence. It is worth noting that the quadratic potential so often used to describe coupling between condensates cannot explain the critical properties of the system, even though the correlation function, Eq. (36), has the same form in both approaches. We conclude from Fig. 2 that particle density is the dominant factor in systems with the density-induced tunneling interaction. The cut-off minimum occurs at the same value of $\mu /U$ as the tip of the superfluid–Mott insulator lobe dominated by the density that locally conserves its integer value. The density induced interaction could be expected to depend strongly on the chemical potential. However, surprisingly, the coherence restored by the density induced tunneling behaves nonmonotonically and in opposition to the critical values of the single particle superfluid of the generic Bose-Hubbard model. The subtlety of the phenomenon should be also noted: the strongest coherence among the bosons is not provided by large densities, but rather small fluctuations thereof. The harmonic coupling model does not provide a valid description for small densities, being almost constant throughout the relevant range of the chemical potential values.

**Figure 3:**
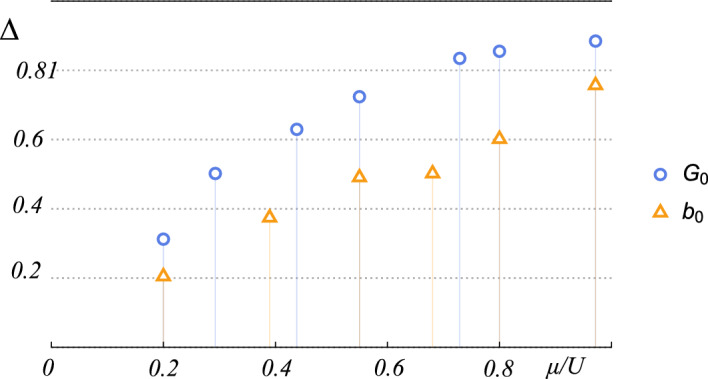
Gap between the first decoherence breakdown and the revival of superfluidity for both models, as indicated in the bottom diagram in Fig. 2.

It is clear that the properties of the system strongly depend on the approach taken, but some features are shared by both of them. The decoherence of the system and the revival of superfluidity are separated by the gap $\Delta$, which monotonically increases with particle density, as shown in Fig. 3. There are no qualitative changes in the gap between both approaches; we conclude that it does not depend on the character of the coupling, but rather on the quantum rotor properties of the critical lines themselves.

**Figure 4:**
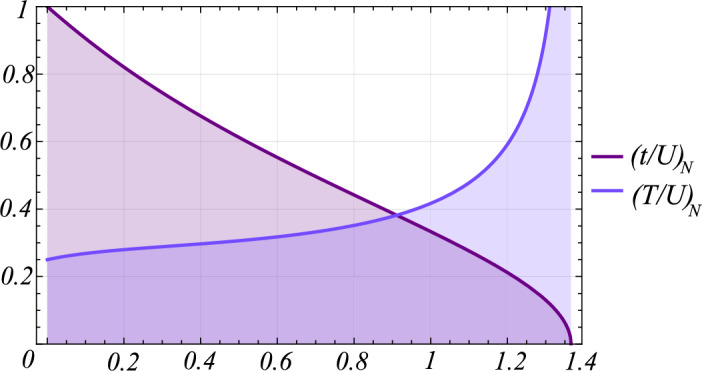
Chemical potential critical behavior of the nearest-neighbor tunneling and density-induced amplitudes, calculated analytically from the critical properties of the phase-phase correlator, Eq. (44) with the upper limit $\mu /U=\left( 1+\sqrt{3}\right) /2\simeq 1.366$. The diagram shows disappearance of both tunneling amplitudes for large condensates densities.

The importance of chemical potential when density-induced tunneling is present led us to analyze the properties of the tunneling amplitudes relative to density. The interesting diagram in Fig. 4 was derived analytically from the phase-phase correlation function in Eq. (44). Increasing particle density has different effects on the nearest-neighbor tunneling *t*/*U* and density-induced tunneling *T*/*U*. The single amplitude *t*/*U* counterintuitively decreases monotonically, with a rather steep decent, and finally goes to zero. In contrast, the DIT stays almost constant, before diverging rapidly to infinity at high densities. The high-density critical behavior of both amplitudes occurs at the same point of $\mu /U=\left( 1+\sqrt{3}\right) /2$. These results suggest that in systems with extended interactions, the chemical potential governs almost all the properties of the system, both diminishing the coherent state and at the same time supporting correlated hopping between bosons. The magnitude of both tunneling amplitudes is equal at $\mu /U=\left( 1+\sqrt{7}\right) /4\simeq 0.91$, where $\left( t/U\right) _{N}=\left( T/U\right) _{N}=\sqrt{\sqrt{7}-5/2}\simeq 0.38$. That provides the boundary of prepotency of density induced interaction.

## Conclusions

In this work, we have shown a quantum rotor analysis of the dissipative aspects of an interaction mediated by particle density within a bosonic system. The density-induced tunneling interaction is known to both affect the single condensate and generate a pair condensed phase. The two condensates can coexist and further affect each other. Our analysis concentrated on the influence of the pair condensed fraction on the single condensate. The system can be driven out of a dissipative state into superfluid. A strong enough DIT interaction can boost coherence among single particles, providing long range order. Taking quantum fluctuations into account leads to decoherence of the system for small values of the density induced term. This is contrary to common beliefs that the aforementioned interaction only supports single particle superfluidity. We also observe an incoherent pair superfluid phase, with no indication of single particle superfluidity within. Observing such phenomena requires including quantum fluctuations in theory and higher values of density induced interaction amplitudes in experiments.

We studied the system using two different approaches. The first approach assumed that the single superfluid is in contact with a harmonic reservoir of pair superfluid. This version provides correct predictions of the dissipative character of the environment; however, it fails at large densities. We thus show that a constructed theory, which assumes harmonic coupling between the two condensates, cannot provide a proper description of the critical behavior of the system, even though it might to some degree take into account quantum fluctuations. The second approach was based on preserving the unabbreviated form of the phase-phase correlator and its imaginary time properties. This version provides a valid description and predicts an unprescribed cutoff of coherence within the single particle superfluid. It is shown that particle density governs the behavior of the system and imposes interchangeable phase transitions. The latter might be easily missed: in theory, by assuming harmonic character of inter–condensate coupling; in experiments, by not controlling the density and amplitude of the density induced interaction.

## Figures and Tables

**Figure 5:**
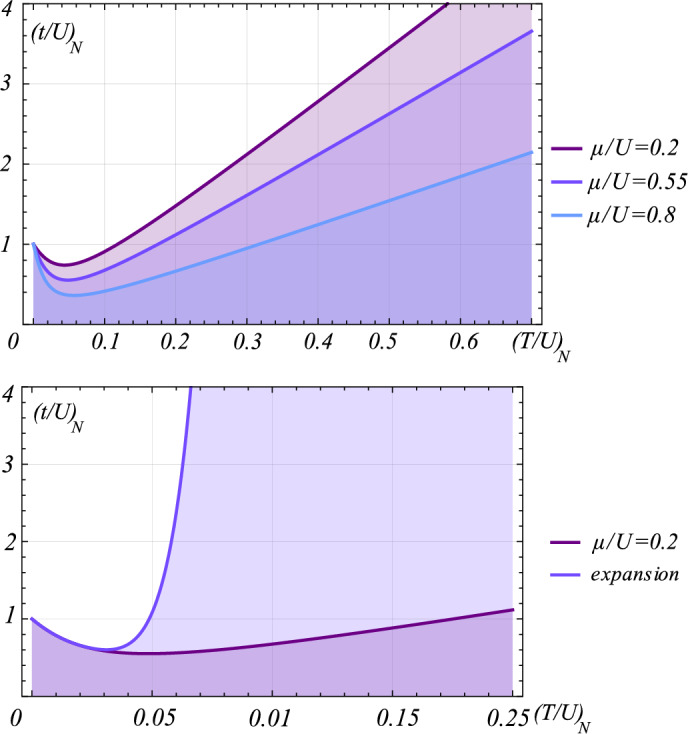
Comparison of the dependence of normalized single hopping $\left( t/U\right) _{N}$ on the normalized DIT coefficient $\left( T/U\right) _{N}$ at different chemical potentials $\mu /U$, resulting from analytical calculations of the critical properties of the phase-phase correlation function Eq. (44). Bottom: analytical model versus expansion, Eq. (43).

**Figure 6:**
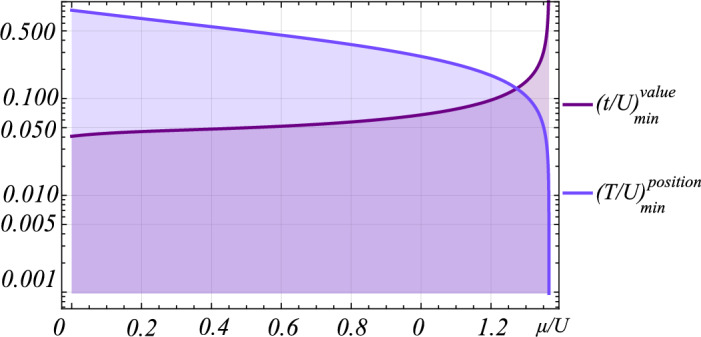
Chemical potential dependence of the position of the minimum density induced $\left( T/U\right) _{\textrm{min}}$ and hopping $\left( t/U\right) _{\textrm{min}}$ amplitudes from Fig. 5.

## Data Availability

All data generated or analyzed during this study are included in this published article.

## Appendix 1: Low temperature properties of the phase-phase correlation function

After Mastubara summation and consequent Fourier transform, the full form of the phase-phase correlator, Eq. (36), can be rewritten as

44 $$\begin{aligned} \mathcal {G}\left( \omega _{m}\right)&=\frac{\exp c}{\sqrt{2\pi }}b^{-\frac{a}{2}-\frac{1}{2}}\Gamma \left( \frac{a+1}{2}\right) \,_{1}F_{1}\left( \frac{a+1}{2};\frac{1}{2};-\frac{\omega _{m}^{2}}{4b}\right) , \end{aligned}$$

where

45 $$\begin{aligned} a&=\frac{2}{8\pi g_{2}}, \end{aligned}$$

46 $$\begin{aligned} b&=\frac{\pi ^{2}}{24\pi \beta ^{2}g_{2}}, \end{aligned}$$

47 $$\begin{aligned} c&=\frac{2H_{\frac{4g_{2}\beta }{\pi }}+2\ln \frac{2\pi }{\beta }-\frac{\pi }{\left( 4\beta g_{2}+\pi \right) }}{8\pi g_{2}}, \end{aligned}$$

$\Gamma$ is the Euler gamma function, $\,_{1}F_{1}$ is the Kummer confluent hypergeometric function, and $H_{n}$ is the $n^{\text {th}}$ harmonic number.

The correlator is convergent as long as $g_{2}<\left( 8\pi \right) ^{-1}$, which corresponds to an upper limit on the chemical potential for both versions of $g_{2}$, Eqs. (20) and (27). Within the relevant range of tunneling parameters *t* and *T*, the upper limit is $\mu /U=\left( 1+\sqrt{3}\right) /2\simeq 1.366$. We focus on low-density systems in order to remain beneath this value. Other properties can also be calculated from the convergence condition, providing analytical results to compare with the numerical data obtained from the critical line equation. Although the amplitudes $g_{0}$ and $G_{0}$ strongly affect the results, within some properties of the system, especially where the quantum fluctuations are very strong, the analytical predictions fit very well with numerical experiments Figs. 5 and 6. The position and value of the minimum of the normalized hopping in Fig. 6 can be derived analytically and yields

48 $$\begin{aligned} \left( {\frac{t}{U}}\right) _{min}^{value}&=\frac{1}{8\sqrt{3\pi }\sqrt{-2\mu ^{2}+2\mu +1}}\left| \frac{4\mu +1}{2\mu +1}\right| ,\end{aligned}$$

49 $$\begin{aligned} \left( {\frac{T}{U}}\right) _{min}^{position}&=\sqrt{\frac{2}{3}}\frac{\sqrt{-2\mu ^{2}+2\mu +1}}{2\mu +1}. \end{aligned}$$

The boundaries of the model parameters can be deduced from this minimum.
